# Supplementary material for: A bibliometric analysis of nasopharyngeal carcinoma radiomics: trends and insights
Source: Front Oncol. 2025 Mar 25;15:1506778. doi: 10.3389/fonc.2025.1506778 (PMC11975905; doi:10.3389/fonc.2025.1506778)
Supplement: Supplementary file 1 [file Table1.docx]

"Nasopharyngeal carcinoma" OR "Nasopharyngeal carcinomas" OR "Nasopharyngeal cancer" OR "Nasopharyngeal cancers" OR "NPC" OR "NPCs" OR "Nasopharyngeal neoplasm" OR "Nasopharyngeal neoplasms" OR "Nasopharyngeal tumor" OR "Nasopharyngeal tumors" OR "Carcinoma of nasopharynx" OR "Cancer of nasopharynx" OR "Carcinomas of nasopharynx" OR "Cancers of nasopharynx"

"Radiomics" OR "Radiogenomics" OR "Radiomics signature" OR "Radiomics model" OR "Radiomic features"OR "Radiomics analysis" OR "Radiomics workflow" OR "Radiomics pipeline" OR "Radiomics studies" OR "Radiomics approach" OR "Radiomics-based" OR "Radiomics data" OR "Imaging genomics" OR "Imaging features" OR "Image analysis" OR "Image-based analysis" OR "Texture analysis" OR "Quantitative imaging"
